# Estimating the prevalence of key healthcare-associated and opportunistic infections in Australian transplant and cancer populations: protocol for the PROSPER point prevalence study

**DOI:** 10.1136/bmjopen-2025-100798

**Published:** 2025-08-01

**Authors:** Priya Garg, Nikhil Singh, Alice J Liu, Michelle K Yong, Monica A Slavin, Lisa Hall, Leon J Worth

**Affiliations:** 1 Department of Infectious Diseases, Peter MacCallum Cancer Centre, Melbourne, Victoria, Australia; 2 National Centre for Infections in Cancer, Peter MacCallum Cancer Centre, Melbourne, Victoria, Australia; 3 Faculty of Medicine Dentistry and Health Sciences, The University of Melbourne, Melbourne, Victoria, Australia; 4 Department of Infectious Diseases, Westmead Hospital, Sydney, NSW, Australia; 5 Victorian Infectious Diseases Service, The Royal Melbourne Hospital, Melbourne, Victoria, Australia; 6 School of Public Health, Faculty of Medicine, The University of Queensland, Brisbane, Queensland, Australia; 7 Victorian Healthcare Associated Infection Surveillance System (VICNISS) Coordinating Centre, The Peter Doherty Institute for Infection and Immunity, Melbourne, Victoria, Australia

**Keywords:** Prevalence, Infection control, INFECTIOUS DISEASES, TRANSPLANT MEDICINE, ONCOLOGY, HAEMATOLOGY

## Abstract

**Abstract:**

**Introduction:**

The immunocompromised host (ICH) is at increased risk for a range of opportunistic and healthcare-associated infections (OI/HAIs). With increased use of novel therapeutics and prolonged survival, the malignancy and transplant population is a particularly vulnerable and expanding subgroup. In the absence of a coordinated Australian infection surveillance programme, estimates of the prevalence of OI/HAIs for the high-risk ICH population are yet to be established. Approaches to infection prevention and control (IPC) are also non-standardised across healthcare facilities (HCFs). This study aims to provide data on key pathogen prevalence and comparative IPC and infection monitoring practice amongst the Australian cancer/transplant population to inform future consensus ICH-specific policy.

**Methods and analysis:**

The first multi-site adapted point prevalence survey (PPS) for OI/HAIs in the high-risk ICH population will be conducted across several Australian public HCFs providing inpatient (IP) care for adult transplant (solid organ/haematopoietic stem-cell)±malignancy (haematological/oncological) patients. Surveillance methodology using the European Centre for Disease Prevention and Control (ECDC) PPS protocol modified for the ICH will be applied. ICH-adapted ECDC and Centres for Disease Control and Prevention (CDC)/National Healthcare Safety Network (NHSN) surveillance case definitions will be used for key HAIs and diagnostic criteria for select OIs. Potentially eligible cancer and transplant patients will be identified for sampling by active antimicrobial use. Infection data, patient-level risks and correlates for HCF impact will be collected from medical records. To contextualise infectious rates, IPC and surveillance strategy will be explored through qualitative interviews with IPC personnel at each sampling site. The prevalence of infection will be approximated from the proportion with infection in the sample screened, and descriptive data analysis will be used to support the expected outcomes of this study, which includes providing a unique insight into infectious disease trends alongside current IPC and surveillance processes within this highly specialised population.

**Ethics and dissemination:**

Ethical approval has been obtained from the Peter MacCallum Cancer Centre (PMCC) Human Research Ethics Committee (HREC/112164/PMCC) via the National Mutual Acceptance Scheme. Research findings will be disseminated through peer-review publication and conference presentation and contribute to future work on consensus IPC and surveillance guidelines.

**STRENGTHS AND LIMITATIONS OF THIS STUDY**

- This will be the first study internationally to describe the prevalence of key opportunistic and healthcare-associated infection (HAI) in the cancer and transplant population using a point prevalence survey approach.
- Surveillance methodology is based on the European Centre for Disease Prevention and Control point prevalence protocol, which provides a validated framework to investigate HAI.
- This protocol represents a distinct opportunity to trial adapted surveillance case definitions specific to the needs of the immunocompromised host.
- The focus on ward-based inpatients and use of antimicrobial therapy as a screening tool may under-estimate true infectious rates.
- Geographic and seasonal variation in infection should be considered when interpreting study data.

## Introduction

 Healthcare-associated infections (HAIs), infections acquired as a direct or indirect result of healthcare, are one of the most common avoidable adverse events in healthcare settings, associated with significant morbidity, mortality and healthcare expenditure.^1^ HAI surveillance is a key component of infection prevention and control (IPC), offering unique epidemiological insights to respond to emerging threats, guide the design of targeted intervention and benchmark results for future comparative analysis.^2^ Point prevalence survey (PPS) methodology provides a validated, well-established framework to capture the burden of disease at a point in time, used regularly by the European Centre for Disease Prevention and Control (ECDC) and Centres for Disease Control and Prevention (CDC) for HAI monitoring across Europe and the USA.^2^,^4^

Australia does not yet have a standardised framework for HAI surveillance, with significant variation in monitoring practices across states, territories and even individual healthcare facilities (HCFs).^4 5^ Healthcare-associated *Staphylococcus aureus* bacteraemia is the only nationally mandated and reported infection indicator.^6^ There have also only been two Australia-wide HAI point prevalence studies since 1984 outside of an Intensive Care Unit (ICU) setting, primarily focused on the general adult hospitalised cohort.^4 7^ Of note, the most recent PPS (CHAINS) excluded specialist hospitals, including cancer centres, from data collection, and patient-level risks were not collected.^8 9^

Cancer and transplant patients are a nationally expanding population, with notable vulnerability to infection.^10^ This is both as a consequence of innate disease-related risk, alongside requirements for often profoundly immunosuppressive therapy, indwelling devices, surgical interventions, broad spectrum antimicrobials and multiple, prolonged healthcare exposures.^1 11 12^ In Australia in 2023 alone, there were an estimated 165 000 new cancer cases and 1396 organ transplant recipients, with almost 2000 haematopoietic stem-cell transplants (HSCTs) performed annually.^13^,^15^ Coupled with immunocompromised host (ICH) population growth, there has also been a surge in the use of novel immune therapies, which have revolutionised the therapeutic landscape, but in the setting of immune modulation, contribute to increased infectious risk.^16^

Internationally, the Hospital Infection Surveillance System for Patients with Haematologic/Oncologic Malignancies (ONKO-KISS), a German/Swiss/Austrian trinational establishment, has historically led selective HAI surveillance within groups of haemato-oncology patients; however, reporting accuracy is limited by the applicability of traditional surveillance case definitions for this population who may present atypically with infection, timeliness in the context of labour-intensive data collection over years and utility in the breadth of data collected.^17^,^19^ Thus, adapted models are called for.^18^

Additionally, although consortiums such as the Swiss Transplant Cohort Study allow for the collection of data on specific opportunistic infections (OIs) (infections occurring with greater frequency or severity in those with compromised immunity) within the solid-organ transplant (SOT) population, to help guide targeted IPC strategy such as screening, prophylaxis and vaccination, comprehensive OI surveillance is also limited by a lack of standardised case definitions in the cancer and transplant setting, and consensus mechanisms for monitoring.^20 21^

We will undertake the first multi-site multi-modal PPS for key HAIs and select locally relevant OIs among the cancer and transplant population in Australia. The latest ECDC protocol (V.6.1) provides a validated framework to survey HAIs within an acute care hospital setting.^22^ This has been tailored to meet the specific needs of the high-risk ICH, including, notably, the capture of OIs, given the heightened susceptibility of the ICH to both OIs/HAIs. Understanding the prevalence of and risk factors for both OI/HAIs among the cancer and transplant population is crucial to identify areas for focused quality improvement to strengthen IPC and surveillance mechanisms, reduce rates of preventable infection, refine prophylactic strategies and optimise ward-based clinical outcomes for the high-risk ICH admitted to Australian HCFs.

Australia lacks both a nationally coordinated system for infectious surveillance and consensus IPC guidelines, with research highlighting clear disparities between jurisdictional methodology.^4 18 23^ Thus, there is a paucity of data on the epidemiology of OIs and HAIs among the Australian hospitalised cancer and transplant population.^18^ This study aims to provide a descriptive insight into rare disease trends within a specialised population.

### Study objectives

The primary objectives of the PROSPER study (Point Prevalence Study of Opportunistic and Healthcare-Associated Infections (OI/HAIs) in Australian Transplant and Cancer Patients) are:

1. Describe the prevalence of key OI/HAIs among the adult (≥18 years) cancer (oncological/haematological) and transplant (HSCT/SOT) population receiving acute inpatient (IP) ward-based care within Australian public HCFs.
2. Describe key OI/HAIs by pathogen type and patient demographic.
3. Describe key OI/HAIs and impact on HCF (bed-days +/− critical care use related to infection during IP admission).

Secondary objectives include describing the prevalence of ICH patients at the time of the PPS:

1. With an indwelling device.
2. Colonised or undergoing treatment for a multidrug-resistant organism (MDRO) infection.
3. On antimicrobial prophylaxis.

Current practice for IPC and surveillance strategy at recruitment sites will also be evaluated to contextualise OI/HAI rates, compare practice and identify areas for strengthening of policy.

## Materials and methods

### Study design

A multi-centre observational PPS will be conducted across a sample of Australian public HCFs with the capacity to provide acute medical ward-based IP care for adult cancer and transplant (HSCT/SOT) patients. Outpatient care facilities are not a prerequisite for study inclusion as these settings will not be surveyed. There is no precedent for a specific PPS protocol to capture both OIs and HAIs in the high-risk ICH. An adapted version of the ECDC PPS protocol has previously been operationalised in the Australian setting.^8^ ICH-appropriate modifications to the ECDC V.6.1 PPS have been made based on input from a focus group of IPC and ICH infectious disease specialists (online supplemental table 1). These adjustments have been made to reflect the differing risk profiles, complex health backgrounds and often atypical clinical presentations of the high-risk ICH compared with the immunocompetent population.

### Hospital selection

Acute care public hospitals with IP facilities for adult HSCT/SOT recipients and/or patients with malignancy have been recruited through expression of interest and convenience sampling, as supported by the ECDC PPS.^22^ Limited numbers of Australian HCFs provide care for all high-risk ICH patients, thus, multiple sites are required to ensure adequate and representative recruitment. Collaboration across centres will also allow for greater patient diversity and demographics, increasing the likelihood of generalisability, yet permitting comparison in variation in IPC practice and OI/HAI prevalence rates. We expect 5 sites will participate, representing the states/settings responsible for the majority of treatment of cancer and transplant patients in Australia.

### Ward selection

In each participating HCF, all acute IP wards will be included except for:

- ICU.
- Hospital-In-The-Home (HITH) services.
- Emergency Departments (ED).
- Palliative or hospice-care wards.

This study aims to inform improvements in ward-based management for the high-risk ICH, thus ICUs will be omitted to ensure relevance to standard IP care environments for cancer/transplant patients in the Australian setting. ICU patients additionally typically have differing levels of care and are more likely to require invasive devices and interventions, all of which may disproportionately influence the prevalence of HAIs.^24^ Reflecting this, dedicated surveys are often performed for HAIs specifically acquired in the ICU, which allow for optimisation of clinical care specific to the ICU context.^25^ Similarly, patients receiving palliative/end-of-life care frequently require pragmatic decision-making regarding use of indwelling devices, antimicrobial therapy as well as regularity of blood and diagnostic tests.^26^ Participants in ED or HITH will also be excluded as, by nature of their healthcare interaction, may not be able to meet surveillance case definitions for HAIs, and in the context of OIs, medical record data may be limited for the determination of OIs.

### Patients sampling and sample size

As per ECDC protocol, all eligible participants will be included in the survey.^22^ As the prevalence of OI/HAIs among this specific population is yet to be determined and OI/HAIs are rare, a reasonable sample size of 50 infected participants per HCF has been preselected to allow for sufficient descriptive analysis of infections to be produced, and for a prevalence estimate of participants with infection over the total eligible population screened to be inferred.^27^ Similar methodology has been used in rare disease and fungal surveillance.^28 29^ Given that the ICH may be admitted to hospital for a multitude of non-infective reasons, it is anticipated that only a small number of patients screened at any single point in time will meet study-specific case definitions for an OI/HAI. Therefore, multiple single-day PPS will be performed at fortnightly intervals until target recruitment at each HCF is achieved. For feasibility, survey completion will be retrospective, with patient-level medical records facilitating the extraction of a hospital-wide IP list for each nominated survey day. A variable duration of retrospect will therefore be expected at each site. The choice of discrete fortnightly sampling points reduces the risk of duplication by maximising IP bed turnover. The total eligible population screened at each site to reach 50 infected cases will form the denominator for prevalence estimation descriptives.

### Patient selection

The following inclusion criteria will be applied:

1. Adult patients ≥18 years.
2. Patients with malignancy (haematological/oncological), HSCT +/− SOT transplant recipients (allogeneic/autologous).
3. Receiving IP ward-based care within an acute-care public HCF.

As per ECDC protocol, patients admitted to an IP ward before or at 08:00 am on the PPS survey day (and not discharged) will be eligible for sampling.^22^ Adult cancer and transplant IP will be identified through a daily census list generated by the medical record system.

Eligible patients to be screened for OI/HAIs will be those receiving or scheduled to receive a therapeutic antimicrobial drug on the survey date. Stratifying patients by antimicrobial therapy is an internationally validated methodological approach performed in large multi-site HAI point prevalence studies.^2^ Alternative methods have involved stratifying patients by the presence of fever (core temperature ≥38.0°C) in conjunction with antimicrobial therapy; however, the ICH cohort may not mount a febrile response to infection.^8 18^ Additionally, patients on appropriate antimicrobial therapy for OI/HAIs may no longer be febrile.

### Data collection/management

The study will aim to commence recruitment at participating sites from early 2025. Data collection will occur using research electronic data capture (REDCap), a secure web-based data management tool. A local site primary investigator (PI) will be appointed based on clinical and research experience to facilitate data collection and protocol implementation. The composition of the data collection team at each site will vary depending on resourcing; however, a local antimicrobial stewardship pharmacist will be recommended to help guide recruitment. All study staff will be provided training in the use of medical records, collection methodology and the data collection tool. Overall support for each site will be provided by the lead and coordinating principal investigator (CPI). This study will collect patient, infection (HAI/OI) and IPC/surveillance data. A robust data management plan will be implemented to ensure that all data obtained during this study will be stored, managed and analysed securely.

### Patient data

Relevant patient-level data will be collected from the medical record to allow investigators to record patient demographics, infectious episodes and describe potential risk factors of interest (table 1).

**Table 1:** Patient data for collection in PROSPER study

Patient demographics	Age, gender as per the medical record Country of birth, occupation
Admission details	Date of hospital admission, admitting medical/surgical speciality Admission type (emergency/elective/readmission/transfer) Intensive care requirement related to infection during inpatient stay (bed-days)
Infection prevention	Multi-drug-resistant organism (MDRO) colonisation status Infection prevention precautions at the time of the survey Indwelling medical devices at the time of the survey
Targeted medical history	Surgical interventions within 90 days of survey (name, date) Absolute neutrophil count on calendar day/day before survey Requirements for phage/Chimeric Antigen Receptor (CAR) T-Cell therapy during admission Malignancy details if relevant: malignancy, disease status (eg, active/in remission), year of first diagnosis, presence of metastases, recent (within 6 months of survey date) malignancy-related therapy Transplant details if relevant: transplant type, transplant number (eg, primary/repeat), donor status, time post most recent transplant, recent (within 6 months of survey date) transplant-related therapy Receipt of annual influenza vaccination
Allergies/antimicrobials	Antimicrobial allergies: drug name, allergy severity (mild/moderate/severe) Antimicrobial prophylaxis on the day of admission (or within 30 days of survey date)

### Infection data/prioritisation

Key OI/HAIs for surveillance in this study have been prioritised according to healthcare cost, anticipated impact on patient outcomes, potential for preventability, access to diagnostic tests and availability of evidence-based antimicrobial therapy, specific to the cancer/transplant population. These have been selected in consultation with a focus group of IPC and ICH infectious disease specialists at the National Centre for Infections in Cancer (NCIC).

### HAI definitions

Key HAIs for surveillance in the PROSPER study are:

- Central-line associated bloodstream infection.
- Catheter-associated urinary tract infection.
- Surgical site infection.
- Clostridioides difficile infection.

Study-specific surveillance case definitions will be applied, using validated international criteria, modified for the ICH.^22 30^ (See online supplemental table 2) for ICH-adapted HAI definitions).

### OI definitions/prioritisation

Key OIs for the study have been defined by the NCIC ICH-specialist steering group for the purposes of this study as ICH-relevant infections with modifiable risk factors (vaccination or prophylaxis), or for which laboratory screening methods are routinely available and regularly used prior to commencing immunosuppressive therapy, and or active antimicrobial therapeutic options exist. (See table 2 for key OIs).

**Table 2:** Select opportunistic infections for the Australian high-risk immunocompromised host for capture in the PROSPER study. Treatment of the infection, as listed below, must be recorded in the medical record, or diagnosis documented as diagnosed by an ID/microbiology specialist or service, whether using laboratory and/or clinical/epidemiological factors

Virus	Bacteria	Fungus	Parasite/other
Cytomegalovirus infection (CMV)	Non-tuberculous mycobacteria spp. (NTM)	All invasive fungal infection receiving antifungal therapy—as determined by an infectious disease/microbiology specialist (Including Pneumocystis Jirovecii pneumonia (PJP)	Strongyloides stercoralis
Varicella zoster virus (VZV)	Mycobacterium tuberculosis (MTB)		Toxoplasma gondii
Hepatitis B	Invasive streptococcus pneumoniae		
Hepatitis C	Neisseria meningitidis (ACYW/B)		
Influenza A/B	Treponema pallidum (syphilis)		
Respiratory syncytial virus (RSV)	Haemophilus influenzae B		
COVID-19			
Herpes simplex virus (HSV-1/2)			

### Infection data collection

The following data will be collected per confirmed OI/HAI episode:

- Type of HAI or clinically diagnosed key OI.
- Date of onset.
- Relevant microbiology (pathogen, major MDRO status where relevant—Methicillin-resistant *Staphylococcus aureus*/Vancomycin-resistant Enterococcus/Extended-spectrum beta-lactamase/Carbapenem-producing Enterobacterales, multidrug-resistant gram-negative bacteria.
- Current antimicrobial therapy.

MDRO status will be documented as defined by the local laboratory responsible for analysing and releasing the specimen result. Given the immunosuppressed status of surveyed populations, each recruited ICH participant may have multiple active infectious episodes. In those cases, each infection will be individually documented under the appropriate participant. This will allow for analysis of the total number of infectious episodes as well as patients with an OI/HAI at any given sampling point.

### IPC/surveillance data collection

Site IPC data collection is part of the ECDC V.6.1 PPS protocol.^22^ Adapted questions for each recruited site, modified for the ICH, have been created.

Data will be obtained through a semi-structured interview with a healthcare worker (HCW) within the local IPC team, comprising basic ICH and IPC demographics for each site and then an exploration of current ICH-IPC and infectious surveillance practice. The most appropriate member of the IPC team at each site will be nominated by each site PI for interview. They will be chosen on the basis of overall experience in IPC/surveillance and years of practice at the HCF. If they choose not to proceed, further nominations of the next most appropriate team member will occur. The consolidated framework for implementation research has been used to guide interview design.^31^

This interview will verify information on:

- High-risk ICH demographics managed at the hospital.
- IPC resourcing (infrastructure, staff/services).

And then explore through open-ended questioning:

- IPC service components.
- Current infectious surveillance systems for OI/HAIs.
- Key areas for future quality improvement.

### Data validation

Data validation exercises will be performed as part of training to ensure a clear understanding of study methods, consistency in surveillance case definition application and data recording. Recorded data will be assessed for completeness and accuracy after collection at the first participating HCF by the lead and CPI, and outcomes addressed through a formal meeting with the study team before commencing at other sites. Each site’s progress will be overseen by the lead investigator and CPI. Where there is ambiguity regarding OI/HAI definitions, this will be resolved in a formal meeting between validation team members (including the site PI, lead investigator and/or CPI) until consensus is achieved.

### Data analysis

Descriptive statistical analyses will be performed. As the true prevalence of OI/HAIs among this population is unknown, a target recruitment of 50 infected cases per site has been pragmatically chosen to sufficiently address the study questions. The prevalence of OI/HAI will be described from the proportion with infection in the total sample screened at each discrete sampling time-point. A broader prevalence estimate will also be drawn from the fixed numerator of participants with infection at each study site, compared with a denominator of all eligible patients screened across time-points and across sites, using aggregated weighted average prevalence. Sensitivity analysis will be applied where appropriate. Data will be expressed as numbers and percentages, means with SD or medians with IQR as appropriate. Differences between HCFs and study participant groups will be analysed using χ^2^ or Fisher’s exact test for categorical parameters and Student’s t-test or Mann-Whitney U test for continuous parameters as appropriate. Similar methodology estimating disease prevalence among a screened population includes rare disease registries and the surveillance of fungal disease.^28 29 32^

Qualitative interview answers will be analysed using interpretative descriptive methodology to explore perspectives on IPC interventions and surveillance practice implemented at each site for the high-risk ICH.^33^ Thematic analysis through coding of open-ended interview responses will allow for identification of broader connections.

### Outcome measures

The main outcome measure will be the estimated prevalence of key OI/HAIs among adult high-risk ICH IPs in public HCFs in the study. Further objectives of this research include describing key OI/HAIs by pathogen type and patient demographic, as well as impact on HCFs (bed-days, and % time spent in critical care related to infection). The prevalence of patients with an indwelling device, and those colonised with or undergoing treatment for an MDRO infection and on antimicrobial prophylaxis at the time of the PPS will also be recorded.

Interview data captured during this study will provide a rich IPC and surveillance context to OI/HAI rates at each HCF. This context will deepen the research team’s interpretation of OI/HAI data collected from each hospital, allow for consensus and heterogeneity in current IPC/infection monitoring practice to be examined across sites, evaluate implementation, identify potential gaps and highlight areas for policy strengthening. An interview format has been used in previously published work to provide a broad insight into the issues guiding the creation of large-scale surveillance programmes from a general perspective.^33^

### Ethical considerations and dissemination

This study is sponsored by the Peter MacCallum Cancer Centre (PMCC) (HREC/112164/PMCC). Ethical considerations have been reviewed by the human research ethics committee (HREC) at the PMCC via the National Mutual Acceptance Scheme, an Australia-wide system for mutual acceptance of scientific and ethical review for multi-centre trials in publicly funded health services.

### Informed consent (Patients)

A waiver of consent for the collection of patient and infection data has been approved for this study. This is based on the following considerations as outlined in the National Health and Medical Research Council National Statement on Ethical Conduct in Human Research (2023 and updates), section 2.3.10: involvement in the research carries low to negligible risk to participants, there is no direct patient interaction or intervention, only existing information available through usual duty of care will be used, study results will have no direct consequence on patient welfare, a robust management plan is in place to protect participant privacy, and the benefits of the research are thought to outweigh any risks of harm from not seeking individualised consent.^34^

### Informed consent (HCWs)

A written participant information sheet denoting study objectives will be provided for the designated IPC HCW at the study site. This will be issued by a named study investigator performing the interview, and the HCW required to provide verbal consent at interview commencement. The HCW may consent and subsequently withdraw at any time prior to study conclusion. HCWs will not, however, be able to edit or alter interview responses once the interview is complete to ensure that the incorporated data accurately reflects IPC and surveillance strategy in place at the time of the PPS and to reduce the risk of alteration bias.

### Dissemination

It is expected that aggregated anonymised data from this survey will be prepared in manuscript form for peer-reviewed publication and/or research presentation. Authorship will be determined in accordance with the International Committee of Medical Journal Editors guidelines.

## Supplementary material

10.1136/bmjopen-2025-100798 online supplemental file 1

10.1136/bmjopen-2025-100798 online supplemental file 2
