# Supplementary material for: Estimating the prevalence of key healthcare-associated and opportunistic infections in Australian transplant and cancer populations: protocol for the PROSPER point prevalence study
Source: BMJ Open. 2025 Aug 1;15(7):e100798. doi: 10.1136/bmjopen-2025-100798 (PMC12314944; doi:10.1136/bmjopen-2025-100798)
Supplement: online supplemental file 1 [file bmjopen-15-7-s001.docx]

**Supplemental Table 1)** **Summary of European Centre for Disease Prevention and Control (ECDC) Point Prevalence Survey of healthcare-associated infections (HAIs) criteria (Protocol Version 6.1) and major areas of variation to methodology for the purposes of the PROSPER study ^22^**

| **ECDC protocol** | **Deviations** | **Rationale** |
| --- | --- | --- |
| **Patient inclusion and exclusion criteria** | | |
| - All patients admitted to the ward before or at 08:00am and not discharged from the ward at the time of survey, including neonates on maternity and paediatric wards, will be included. | - Only adults ≥18 years old with active malignancy (haematological/ oncological) and or haematopoietic stem cell transplant recipients (allogeneic/ autologous)/ solid organ transplant recipients admitted before or at 08:00am and not discharged from the ward at the time of survey will be included - ICU, HITH, palliative care and ED patients will be excluded | - Specialised patient cohort (patients with malignancy and or transplant recipients) - Paediatric patients to be excluded given differences in epidemiology, treatment and definitions for infection in this cohort compared to adults - ICU, palliative care patients will be excluded given their underlying disease status requiring a specialised model of care compared to the cancer/ transplant patient admitted to a general acute care ward - HITH and ED excluded given nature of healthcare interaction |
| **Data collection processes** | | |
| - Data to be collected in a single day for each ward/unit. Total time frame for data collection for all wards of a single hospital did not exceed 2 to 3 weeks | - We will conduct retrospective point prevalence studies until 50 patients with infection are identified at each site. The number of patients screened will be used to provide an estimate of prevalence. Therefore, a variable duration of retrospect will be expected | - OI/HAIs are rare but significant infections amongst a specialised population, thus multiple retrospective studies at discrete time points will be used to ensure adequate patient recruitment for descriptive analysis - Data from a single day will not provide sufficient numbers - Pragmatic decision based on existing resources and data collection team - Adapted methodology to be considered in appropriate data analysis |
| - ECDC PPS protocol uses existing European case definitions and complements them by case definitions from the CDC as used by CDC’s NHSN | - ICH-adapted ECDC/CDC NHSN case definitions to be used to define HAIs | - The ICH is a specialised cohort amongst whom infection may present atypically, thus modification of existing ECDC/CDC NHSN case definitions through specialist focus group review has occurred to ensure appropriate data capture |
|  | - Select OIs to be captured through diagnostic definitions. There is no precedent for definitions of OIs for cancer and transplant, therefore these will be defined for this study as those with modifiable risk factors (vaccination and or antimicrobial prophylaxis) or that for which screening is generally recommended prior to immunosuppressive therapy, and or therapeutic treatment options exist | - This PPS will be supplemented by the capture of key OIs, which are significant and locally relevant to the high-risk ICH patient cohort. There is no existing surveillance case definition for these infections as a collective, thus for feasibility, diagnostic definitions will be used as per protocol |
| **Hospital & Ward/ Patient data fields** | | |
| - McCabe score employed to classify the severity of disease | - Alternative mechanisms of classifying underlying major medical conditions (cancer and or transplant) severity will be used through the capture of patient demographic data | - Severity of underlying cancer and or transplant will be determined using other ICH-specific metrics including transplant status (primary/ secondary), immunosuppression and or chemotherapy received |
| - Patient level data collection | - Patient level data collection has been modified to adapt to the specific needs of the high-risk ICH | - Patient level data collection has been tailored to collect data which will allow for the collection of possible modifiable and non-modifiable risk factors for OIs and HAIs in the ICH, which may help guide future IPC and surveillance strategy |
| - Hospital/ward level data collection | - The nature of data collected will be tailored to the types of infection prevention and control and surveillance methods used in the care of the specific ICH cohort through an in-depth semi-structured interview | - Hospital/ ward level data collection has been tailored to an interview collect data which will allow for the contextualisation of OI/HAI disease in the high-risk ICH |
| **Data validation** | | |
| - Validation of PPS data performed by validation teams visiting a subset of participating hospitals and re-examining a sample of patient files | - Validation team members will consist of the CPI/ lead investigator and site primary investigator or delegate who will formally cross-check site data if required - Internal validation and validation exercises will be performed prior to commencing study | - Pragmatic validation within existing resources |
| - Blinded validation suggested | - Validation team will not be blinded | - Not feasible for this study |
