# Supplementary material for: Estimating the prevalence of key healthcare-associated and opportunistic infections in Australian transplant and cancer populations: protocol for the PROSPER point prevalence study
Source: BMJ Open. 2025 Aug 1;15(7):e100798. doi: 10.1136/bmjopen-2025-100798 (PMC12314944; doi:10.1136/bmjopen-2025-100798)
Supplement: online supplemental file 2 [file bmjopen-15-7-s002.docx]

**Supplemental Table 2) Immunocompromised host (ICH)-adapted Centers for Disease Control and Prevention (CDC)/ National Health and Safety Network (NHSN)/ European Centre for Disease Prevention and Control (ECDC) surveillance case definitions for PROSPER-study key healthcare-associated infections (HAIs)**

| **HAI** | **ICH-adapted surveillance case definition** |
| --- | --- |
| Catheter-line associated blood-stream infection (CLABSI) | Laboratory confirmed bloodstream infection (LCBI) where an eligible central line was present the day the first positive peripheral blood culture was taken or in the 24 hours before (and the central line must also have been in situ for more than 2 consecutive days)  **PLUS**  The bloodstream infection (BSI) is not thought to be related to infection at another site  **AND**  (Mucosal-barrier injury) MBI-LCBI has been excluded (See PROSPER Study adapted MBI-BSI criteria below)  *(Laboratory confirmed BSI are those with a recognised bacterial or fungal pathogen identified from one or more blood specimens through culture, or to genus or species level by non-culture-based methods)*  **PROSPER Study adapted MBI-LCBI Criteria:**  A patient who has been neutropenic (defined as neutrophils <500 cells/mm) on at least 2 separate days within a 7-day period, which includes the collection day of the positive blood specimen, the 3 calendar days before and the 3 calendar days after  **OR** has had an allogeneic haematopoietic stem cell transplant (HSCT) within the last year and currently has severe (Grade III/IV) graft versus host disease (GvHD) as documented by the treating team during this admission in the medical records  **OR** has had diarrhoea with onset on or within the 7 calendar days before the date the positive blood culture was collected. (Diarrhoea as defined by the patient team during the admission in the medical records)  **AND** patient meets organism criteria for MBI-LCBI (isolation of only intestinal organisms from the CDC/NHSN MBI organism list)  **AND**  Healthcare-associated CLABSI is defined as infection occurring on hospital day 3 or later (where admission to hospital = day 1) **OR** the patient presented with infection but was readmitted ≤ 48 hours after same-day or multi-day admission to a healthcare facility **OR** the invasive device was placed on admission day 1 or day 2 leading to an infection before day 3 |
| Catheter-associated urinary tract infection (CAUTI) | Urinary tract infection (UTI) where an indwelling urinary catheter (IUC) was present for any portion of the calendar day on the day of the event or in the 24 hours before (and must have been in situ for more than 2 consecutive days)  **PLUS**  Current infective episode is not thought to be related to infection at another site  **PLUS**  Patient has a positive urine culture with no more than two species of organisms identified, at least one of which is a bacterium of ≥10^5^ CFU/ml  **OR** ≤10^5^ colonies/ml of a single uropathogen (Gram-negative bacteria or *S. saprophyticus*) in a patient being treated with effective antimicrobial agent for a urinary infection.  All elements of the above criterion must occur during the infection window period (7 days including the date of collection of the positive sample itself, the 3-calendar days before and the 3-calendar days after)  **AND**  Healthcare-associated CAUTI is defined as infection occurring on hospital day 3 or later (where admission to hospital = day 1) **OR** the patient presented with infection but was readmitted ≤ 48 hours after same-day or multi-day admission to a healthcare facility **OR** the invasive device was placed on admission day 1 or day 2 leading to an infection before day 3 |
| Surgical site infection (SSI) | 1. **A) For a superficial SSI (SSI-S), patients must meet the following criteria:**   Date of event occurs within 30 days following the operative procedure (where day 1 = last relevant procedure date)  **AND**  Involves only skin and subcutaneous tissue of the incision  **AND**  **The patient has at least one of the following:**   - Purulent drainage from the superficial incision - Organisms identified from an aseptically obtained specimen from the superficial incision or subcutaneous tissue by a culture or non-culture based microbiologic testing method which is performed for the purposes of clinical diagnosis or treatment - Diagnosis of superficial incisional SSI made by the treating surgeon or a specialist ID physician   **B) For a deep-space SSI (SSI-D) patients must meet the following criteria:**  Date of event occurs within 30 days of last surgical intervention if no implant remains in place, or within 90 days if an implant remains in place (where day 1 = last relevant procedure date)  **AND**  Involves deep tissues of the incision (e.g. fascial and muscle layers)  **AND**  **The patient has at least one of the following:**   - Purulent drainage from the deep incision - A deep incision that is deliberately opened or aspirated by a surgeon, physician or physician designee or spontaneously dehisces **AND** organisms identified from the deep soft tissues of the incision by a culture or non-culture based microbiologic testing method which is performed for the purposes of clinical diagnosis or treatment - An abscess or other evidence of infection involving the deep incision detected on gross anatomical exam, histopathological exam or imaging test.  1. **C) For an organ/space SSI (SSI-O) patients must meet the following criteria:**   Infection occurs within 30 days after the operation if no implant is left in place, or within 90 days if an implant is in place  **AND**  The infection appears to be related to the operation and involves any part of the body deeper than the fascial/muscle layers that is opened or manipulated during the operative procedure  **AND**  **The patient has at least one of the following:**   - Purulent drainage from a drain placed into the organ/space - Organism(s) identified from fluid or tissue in the organ/ space by a culture or non-culture based microbiological testing method which is performed for purposes of clinical diagnoses or treatment - An abscess or other evidence of infection involving the organ/space detected on gross anatomical exam, histopathological exam or imaging testing definitive or equivocal for infection   *A surgical implant is a man-made medical device that is surgically implanted into the body during a procedure to replace a missing biological structure, support a damaged biological structure, or enhance an existing biological structure. |
| Clostridioides difficile infection | A Clostridioides difficile (C. difficile) infection (CDI) must meet at least one of the following criteria:  A positive laboratory test result for C. difficile toxin A and/or B, (includes molecular assays; polymerase chain reaction (PCR) and/or toxin assays) tested on an unformed stool specimen  **OR**  A toxin-producing C. difficile organism detected by culture or other laboratory means performed on an unformed stool sample  **AND at least one of the following (without clear alternative cause):**   - Diarrhoeal stools - Toxic megacolon - Pseudomembranous colitis revealed by lower gastro-intestinal endoscopy - Colonic histopathology characteristic of C. difficile infection (with or without diarrhoea) on a specimen obtained during endoscopy or colectomy   **AND**  Healthcare associated CDI is defined as occurring ≥ 48 hours from inpatient admission, and anywhere in the 28-day period after hospital discharge from any inpatient hospital admission  **AND**  The C. difficile toxin-positive laboratory result does not follow a previous C. difficile toxin-positive laboratory result within 14 days even across calendar months and readmissions to the same facility location. (The date of specimen collection of the previous positive C. difficile result is considered Day 1) |
