# Supplementary material for: Estimating the prevalence of key healthcare-associated and opportunistic infections in Australian transplant and cancer populations: protocol for the PROSPER point prevalence study
Source: BMJ Open. 2025 Aug 1;15(7):e100798. doi: 10.1136/bmjopen-2025-100798 (PMC12314944; doi:10.1136/bmjopen-2025-100798)
Supplement: Supplementary file 3 [file bmjopen-2025-100798.reviewer_comments.pdf]

PEER REVIEW HISTORY

BMJ Open publishes all reviews undertaken for accepted manuscripts. Reviewers are asked to complete a checklist review form and are provided with free text boxes to elaborate on their assessment. These free text comments are reproduced below.

ARTICLE DETAILS

Title (Provisional)

Estimating the prevalence of key healthcare associated and opportunistic infections in Australian transplant and cancer populations: Protocol for the PROSPER point prevalence study

Authors

Garg, Priya; Singh, Nikhil; Liu, Alice J; Yong, Michelle; Slavin, Monica; Hall, Lisa; Worth, Leon

---

VERSION 1 - REVIEW

**Reviewer**	**1**
**Name**	**FLORENTIN, Arnaud**
**Affiliation**	**Université de Lorraine, EA4360 APEMAC**
**Date**	**19-Mar-2025**
**COI**	**None**

Thanks for the opportunity to review this paper about the prevalence of HAI and OI in Australian specific patients.

The paper is well-written, but suffers from several abbreviations which are not defined in the text on first use.

It could be interesting to discuss the difference between HAI and OI in the introduction, as well as the interest to survey OI.

In methods, it's sometimes quite difficult to understand the choice made between ECDC and CDC protocols. And why?

I don't understand the exclusion of ICU wards. In ECDC PPS, ICU wards are included. Only accident and emergency departments. Other surveys are dedicated to ICU wards, but it isn't excluded from European PPS.

Why did you choose to only include 4 HAI? You did the work, so all the HAI could be included.

The choice of OI has to be explained more clearly.

**Reviewer**	**2**
**Name**	**Manjurano, Alphaxard**
**Affiliation**	**National Institute for Medical Research Mwanza Research Centre**
**Date**	**18-Jun-2025**
**COI**	**None**

Title: Estimating the prevalence of key healthcare associated and opportunistic infections in Australian transplant and cancer populations: Protocol for the PROSPER point prevalence study

This study aims to provide data on key pathogen prevalence and comparative IPC and infection monitoring practice to inform future consensus ICH-specific policy.

The design and methodology in general do not make chloronological order. On the other hand, I found some of the description of the paper to be well explained, while the description of some important points is missing. Therefore, I recommend that revisions are needed to address some of the issues raised below.

Major comment

- The abstract need to be revised for easy to follow by the reader. Although background is included however some components are not clearly stated such as rationale of the study, the study population details, data analysis plan and expected outcome. The limitation of the study may not be necessary here however if deemed necessary it need to be summarized.
- The contents in the heading and subheading do not match with heading and subheading hence rearrangement is needed.
- There are several acronyms used for the first time without being stated in full.

Minor comments.

- Line 54 page 3: the subheading ‘’ **methods and analysis”**. The contents in the subheading include: study site, design, sample size, recruitment etc hence the heading should read ‘’ **Materials and Methods”.**
- Lines 57-60 page 4; the authors should provide a convincing argument on the sample size used. If the prevalence is unknown in the study area I suggest the authors to refer to other work somewhere.
- **Study design;** more information is required describing the hospital setting and its capacity for patients caring (both inpatients and outpatients)
- Line 55 page 4: the subheading “sample size/methodology’’. The contents in the subheading include sample size and patients’ recruitment’’ Therefore should be changed to read’’ **Patients sampling and sample size**’’.
- Lines 35-42 page 8- the information here should be under background.
- Table 1 page 12 the timing indicated is not clear if it is pm or am?

VERSION 1 - AUTHOR RESPONSE

Reviewer 1

Comment 1. Thanks for the opportunity to review this paper about the prevalence of HAI and OI in Australian specific patients. The paper is well-written but suffers from several abbreviations which are not defined in the text on first use.

Response: Many thanks to the reviewer drawing our attention to this. We have re-reviewed the paper and ensured that all abbreviations are now defined in the main body of text on first use. Please see changes to abbreviation definitions highlighted accordingly in yellow in the revised manuscript *(pages 2-9 from Introduction onwards).*

Comment 2. It could be interesting to discuss the difference between HAI and OI in the introduction, as well as the interest to survey OI.

Response: Many thanks to the reviewer for this useful suggestion. We have incorporated this advice into the introduction in the main body of the paper as suggested (pages 2-3).

The sentence on HAIs now reads (page 2):

‘Healthcare-associated infections (HAIs), infections acquired as a direct or indirect result of healthcare, are one of the most common avoidable adverse events in healthcare settings, associated with significant morbidity, mortality and healthcare expenditure.’

The section on OIs now reads (page 3):

‘Additionally, although consortiums such as the Swiss Transplant Cohort Study (STCS) allow for the collection of data on specific opportunistic infections (OIs) (infections occurring with greater frequency or severity in those with compromised immunity) within the solid-organ transplant (SOT) population, to help guide targetted infection prevention and control (IPC) strategy such as screening, prophylaxis and

vaccination, comprehensive OI surveillance is also limited by a lack of standardised case definitions in the cancer and transplant setting, and consensus mechanisms for monitoring'.

Furthermore, the interest to survey OIs in this study has also been expanded on in the second to last paragraph of the introduction which now reads (page 3);

'The latest ECDC protocol (Version 6.1) provides a validated framework to survey HAIs within an acute care hospital setting. This has been tailored to meet the specific needs of the high-risk ICH, including, notably, the capture of OIs, given the heightened susceptibility of the ICH to both OIs/HAIs. Understanding the prevalence of and risk factors for both OI/HAIs amongst the cancer and transplant population is crucial to identify areas for focussed quality improvement to strengthen IPC and surveillance mechanisms, reduce rates of preventable infection, refine prophylactic strategies and optimise ward-based clinical outcomes for the high-risk ICH admitted to Australian HCFs.'

Comment 3: In methods, it's sometimes quite difficult to understand the choice made between ECDC and CDC protocols. And why?

Response: Many thanks to the reviewer for these helpful comments.

An adapted version of the ECDC PPS protocol has been previously successfully operationalised in the Australian setting (CHAINS) within an immunocompetent host population. Thus, ECDC point prevalence survey methodology was similarly used as the backbone of this Australian study.

Minor adaptations have however been made to tailor this protocol to the cancer/transplant population being surveyed, primarily in the stratification of patients by use of antimicrobial therapy, in lieu of using fever to identify possible patients with infection, reflecting the fact that immunocompromised hosts may not mount a febrile response to infection, and slight modification of existing HAI surveillance definitions for the ICH. This approach was taken to optimise clinical relevance and contextual applicability within the study setting, while preserving alignment with internationally recognised surveillance standards.

To remove any confusion and enhance readability, we have now grouped the limited instances where the CDC protocol might have been referred to under 'internationally validated criteria', whilst maintaining appropriate references.

The relevant sections in methods therefore now read (page 6):

'Stratifying patients by antimicrobial therapy is an internationally validated methodological approach employed in large multi-site HAI point prevalence studies. (2)'

And (page 7)

'Study-specific surveillance case-definitions will be applied, using validated international criteria, modified for the ICH. (See Supplemental Table 2 ICH-adapted HAI definitions) (21, 25)'

Comment 4: I don't understand the exclusion of ICU wards. In ECDC PPS, ICU wards are included. Only accident and emergency departments. Other surveys are dedicated to ICU wards, but it isn't excluded from European PPS.

Response: Many thanks to the reviewer for these important points.

As this study aims to inform improvements in ward-based management and care for the high-risk immunocompromised host, ICUs were therefore omitted to ensure relevance to standard inpatient care environments.

Australian ICUs also provide services for a broad range of mixed medical and surgical patient populations rather than single-specialty units, and a considerable level of medical management is performed on the ward within the Australasian setting for acutely unwell oncology/transplant patients rather than in ICU, thus, their inclusion would likely have provided limited additional numbers of high-risk ICH patients.

The study purpose has now been more clearly explained within our manuscript. We have also removed the phrase 'complex levels of care' from the sentence regarding ICU patients, to reflect that within our setting, complex patients may still be cared for within the cancer/transplant ward environment.

We have further removed reference to the ECDC PPS, and data capture within the ICU setting as indicated by the reviewer and instead simply stated that other surveys are dedicated to ICU wards, which allow for clinical optimisation specific to the intensive care environment.

This section now reads (page 5);

'This study aims to inform improvements in ward-based management for the high-risk ICH, thus ICUs will be omitted to ensure relevance to standard inpatient care environments for cancer/transplant patients in the Australian setting. ICU patients additionally typically have differing levels of care and are more likely to require invasive devices and interventions, all of which may disproportionately influence the prevalence of HAIs. (22) Reflecting this, dedicated surveys are often performed for HAIs specifically acquired in the ICU, which allow for optimisation of clinical care specific to the ICU context.' (23)

We have also further emphasised our study purpose to enhance ward-based care for the cancer/transplant population within our introduction which now reads (page 3);

'Understanding the prevalence of and risk factors for both OI/HAIs amongst the cancer and transplant population is crucial to identify areas for focussed quality improvement to strengthen IPC and surveillance mechanisms, reduce rates of preventable infection, refine prophylactic strategies and optimise ward-based clinical outcomes for the high-risk ICH admitted to Australian HCFs.'

And in our first study objective which now reads (page 3);

The primary objectives of the PROSPER study (Point Prevalence Study of Opportunistic and Healthcare-Associated Infections in Australian Transplant and Cancer Patients) are:

1. Describe the prevalence of key opportunistic and healthcare-associated infections (OI/HAIs) amongst the adult (≥18 years) cancer (oncological/haematological) and transplant (haematopoietic stem-cell transplant (HSCT)/ solid-organ transplant (SOT)) population receiving acute inpatient (IP) ward-based care within Australian public HCFs

Comment 5: Why did you choose to only include 4 HAI? You did the work, so all the HAI could be included. The choice of OI has to be explained more clearly.

Response: Many thanks to the reviewer for their comments and for the opportunity to expand on this further.

Key OIs and HAIs for surveillance in this study have been prioritised according to healthcare cost, anticipated impact on patient outcomes, potential for preventability, access to diagnostic tests and availability of evidence-based antimicrobial therapy, specific to the cancer/transplant population. These have been selected in consultation with a focus group of infection prevention and control and immunocompromised host infectious disease specialists at the National Centre for Infections in Cancer (NCIC).

Key OIs for the study been defined for the purposes of this study as immunocompromised host relevant infections with modifiable risk factors (vaccination or prophylaxis), or for which laboratory screening methods are routinely available and regularly utilised prior to commencing immunosuppressive therapy, and or active antimicrobial therapeutic options exist.

Following this, 4 HAIs were prioritised and 17 OIs.

We have now made the National Centre for Infections in Cancer (NCIC) steering group selection process clearer within the text, and the selection process with reference to the clinical needs of the immunocompromised host. The subheading has also been changed to 'Infection Data/Prioritisation' rather than just 'Infection Data' above HAIs to signpost the process of where and how these specific infections were chosen, and 'OI Definitions/ Prioritisation' as a subheading rather than just 'OI Definitions'.

This section now reads (page 6):

Infection Data/ Prioritisation

'Key OI/HAIs for surveillance in this study have been prioritised according to healthcare cost, anticipated impact on patient outcomes, potential for preventability, access to diagnostic tests and availability of evidence-based antimicrobial therapy, specific to the cancer/transplant population. These have been selected in consultation with a focus group of IPC and ICH infectious disease specialists at the National Centre for Infections in Cancer (NCIC).'

And (page 7):

OI Definitions/ Prioritisation

'Key OIs for the study been defined by the NCIC ICH-specialist steering group for the purposes of this study as ICH-relevant infections with modifiable risk factors (vaccination or prophylaxis), or for which laboratory screening methods are routinely available and regularly utilised prior to commencing immunosuppressive therapy, and or active antimicrobial therapeutic options exist. (See Table 2 for key OIs).'

Reviewer 2

Comment 1: The design and methodology in general do not make chronological order.

Response: Many thanks to the reviewer for the opportunity to expand on this further. We used the template layout and headings of previously published point prevalence protocol papers within the BMJ Open to help guide the formatting of our paper and headings used within ours for design and methodology.

We however appreciate the feedback and have addressed the comments below raised by the reviewer which have greatly assisted in improving the structure of the paper through rewording of headings and subheadings where recommended, and thus improving the order.

(Please see changes made in response to reviewer comments below)

Comment 2. The abstract need to be revised for easy to follow by the reader. Although background is included however some components are not clearly stated such as rationale of the study, the study population details, data analysis plan and expected outcome.

Response: Many thanks to the reviewer for these helpful comments.

As suggested to ensure the abstract is easier to follow by the reader, we have more clearly explained and signposted 1) the rationale/ aims of the study and 2) population details under the 'Introduction' subheading. The study population has also been highlighted in the 'methods and analysis' subheading of the abstract.

We have also more clearly stated the 3) data analysis plan and 4) expected outcome within the 'methods and analysis' section of the abstract. We believe these changes will now help convey the message of the abstract more clearly as recommended.

The abstract 'Introduction' now reads:

Introduction

'The immunocompromised host (ICH) is at increased risk for a range of opportunistic and healthcare-associated infections (OI/HAIs). With increased use of novel therapeutics and prolonged survival, the malignancy and transplant population are a particularly vulnerable and expanding sub-group. In the absence of a coordinated Australian infection surveillance programme, estimates of the prevalence of OI/HAIs for the high-risk ICH population are yet to be established. Approaches to infection prevention and control (IPC) are also non-standardised across healthcare facilities (HCFs). This study aims to provide data on key pathogen prevalence and comparative IPC and infection monitoring practice amongst the Australian cancer/transplant population, to inform future consensus ICH-specific policy.'

The abstract 'Methods and analysis' now reads:

Methods and analysis

'The first multi-site adapted point prevalence survey (PPS) for OI/HAIs in the high-risk ICH population will be conducted across several Australian public HCFs providing inpatient (IP) care for adult transplant (solid organ/haematopoietic stem-cell) +/- malignancy (haematological/ oncological) patients. Surveillance methodology using the European Centre for Disease Prevention and Control (ECDC) PPS protocol modified for the ICH will be applied. ICH-adapted ECDC and Centers for Disease Control and Prevention (CDC)/ National Healthcare Safety Network (NHSN) surveillance case definitions will be used for key healthcare-associated infections (HAIs) and diagnostic criteria for select opportunistic infections (OIs). Potentially eligible transplant and cancer patients will be identified for sampling by active antimicrobial use. Infection data, patient-level risks and correlates for HCF impact will be collected from medical records. To contextualise infectious rates, IPC and surveillance strategy will be explored through qualitative interview with IPC personnel at each sampling site. The prevalence of infection will be approximated from the proportion with infection in the sample screened, and descriptive

data analysis used to support the expected outcomes of this study which includes providing a unique insight into infectious disease trends alongside current IPC and surveillance processes within this highly specialised population.'

Comment 3. The limitation of the study may not be necessary here however if deemed necessary it need to be summarized.

Response: Many thanks to the reviewer for raising this. 5 strengths and limitations had been included as bullet points as per the journal's author guidelines for the paper. We have however incorporated this feedback into this section and summarised this section for enhanced readability per the reviewer's suggestion, paying particular attention to abridging the limitations of the study in this section.

This section now reads:

- This will be the first study internationally to describe the prevalence of key opportunistic and healthcare-associated infection in the cancer and transplant population using a point prevalence survey approach.
- Surveillance methodology is based on the ECDC point prevalence protocol, which provides a validated framework to investigate healthcare associated infection prevalence.
- This protocol represents a distinct opportunity to trial adapted surveillance case definitions specific to the needs of the immunocompromised host.
- The focus on ward-based inpatients and use of antimicrobial therapy as a screening tool may under-estimate true infectious rates.
- Geographic and seasonal variation in infection should be considered when interpreting study data.

Comment 4: The contents in the heading and subheading do not match with heading and subheading hence rearrangement is needed.

Response: Many thanks to the reviewer for noting this. As mentioned above, we have used the layout and headings of the previously published protocol papers within the BMJ Open to help guide the formatting of our paper and headings used.

However, to address this point further, we have reviewed our paper, and rearranged the headings and subheadings as recommended by the reviewer in further comments below. We have also added in/ rephrased certain subheadings including 'Infection Data/Prioritisation', 'OI Definitions/Prioritisation' and 'Infection Data Collection' to assist with clarity and ensure contents are more reflective of their headings. We believe that these rearrangements have significantly enhanced the paper's readability as recommended.

(Please see changes to headings/ subheadings in manuscript as recommended)

Comment 5: There are several acronyms used for the first time without being stated in full.

Response: Many thanks to the reviewer drawing our attention to this. We have re-reviewed the paper and ensured that all abbreviations are now defined in the main body of text on first use. Please see changes to abbreviation definitions highlighted accordingly in yellow in the revised manuscript *(pages 2-9 from Introduction onwards).*

Comment 6: Line 54 page 3: the subheading '' methods and analysis''. The contents in the subheading include study site, design, sample size, recruitment etc hence the

heading should read ‘’ Materials and Methods”

Response: Many thanks. We have altered this as recommended. *(Please see subheading page 4)*

Comment 7: Lines 57-60 page 4; the authors should provide a convincing argument on the sample size used. If the prevalence is unknown in the study area I suggest the authors to refer to other work somewhere.

Response: Many thanks to the reviewer for the opportunity to expand on this.

The prevalence of opportunistic and healthcare associated infections has never been studied using point prevalence methodology within the Australian adult cancer/transplant population. Thus, the prevalence of these infections is unknown. As OI/HAIs are rare, we will conduct point prevalence studies until 50 cases are identified per site (with 5 sites anticipated, providing 250 infectious cases overall). The total number of patients screened will provide the prevalence. Similar methodology has been used in rare disease and fungal infection surveillance. The aim of this study is to provide a descriptive insight into disease trends and this number we believe is sufficiently large to generate meaningful descriptive data.

Similar methodology where a fixed numerator has been used to describe the prevalence of disease in a population that is not well enumerated includes the use of population-based registries for rare infections. (*Nguengang Wakap S, Lambert DM, Olry A, Rodwell C, Gueydan C, Lanneau V, Murphy D, Le Cam Y, Rath A. Estimating cumulative point prevalence of rare diseases: analysis of the Orphanet database. Eur J Hum Genet. 2020 Feb;28(2):165-173. doi: 10.1038/s41431-019-0508-0. Epub 2019 Sep 16. PMID: 31527858; PMCID: PMC6974615.)*

And in the surveillance of fungal disease, to estimate prevalence: (*Bongomin, F.; Gago, S.; Oladele, R.O.; Denning, D.W. Global and Multi-National Prevalence of Fungal Diseases—Estimate Precision. J. Fungi* ***2017****, 3, 57.* https://doi.org/10.3390/jof3040057).

Other studies which have used a sample of approximately 50 infected cases to generate meaningful descriptive infection data within a similar host population includes a recent retrospective cohort study of early initiation of combined therapy for COVID-19 in the severely immunocompromised: (*Rotundo, S., Berardelli, L., Gullì, S. et al. Early initiation of combined therapy in severely immunocompromised patients with COVID-19: a retrospective cohort study. BMC Infect Dis* ***24****, 564 (2024).* https://doi.org/10.1186/s12879-024-09466-y*).*

We have now added in the 3 references above and expanded on our explanation under the ‘sample size’ section of our manuscript (*please see page 5)*.

This section now reads;

‘As per ECDC protocol, all eligible participants will be included in the survey. (21) As the prevalence of OI/HAIs amongst this specific population is yet to be determined and OI/HAIs are rare, a reasonable sample size of 50 infected participants per HCF has been pre-selected to allow for sufficient descriptive analysis of infections to be produced, and for a prevalence estimate of participants with infection over total eligible population screened to be inferred. (25) Similar methodology has been used in rare disease and fungal surveillance. (26, 27) Given the ICH may be admitted to hospital for a multitude of non-infective reasons it is anticipated that only a small number of patients screened at any single point in time will meet study-specific case definitions for an OI/HAI, therefore, multiple single-day PPS will be performed at fortnightly intervals until target recruitment at each HCF is achieved. For feasibility, survey completion will be retrospective, with patient-level medical records facilitating the extraction of a hospital-wide inpatient list for each nominated survey day. A variable duration of retrospect will therefore

be expected at each site. The choice of discrete fortnightly sampling points reduces the risk of duplication by maximising for inpatient bed turnover. The total eligible population screened at each site to reach 50 infected cases will form the denominator for prevalence estimation descriptives.'

Comment 8: Study design; more information is required describing the hospital setting and its capacity for patients caring (both inpatients and outpatients).

Response: Many thanks to the reviewer for this comment and for the opportunity to enhance this section of our manuscript. We have now provided more information to describe the hospital setting and its capacity for patient caring (both inpatients and outpatients).

This section now reads (page 4)

'A multi-centre observational PPS will be conducted across a sample of Australian public HCFs with the capacity to provide acute medical ward-based IP care for adult cancer and transplant (HSCT/SOT) patients. Outpatient care facilities are not a prequisite for study inclusion as these settings will not be surveyed.'

Comment 9: Line 55 page 4: the subheading "sample size/methodology''. The contents in the subheading include sample size and patients' recruitment'' Therefore should be changed to read'' Patients sampling and sample size''.

Response: Many thanks to the reviewer for this suggestion. We have made the change as recommended.

(*Please see page 5 subheading)*

Comment 10: Lines 35-42 page 8- the information here should be under background.

Response: Many thanks to the reviewer for highlighting this. This section has been moved to be under background as recommended.

(Section moved to page 3)

Comment 11: Table 1 page 12 the timing indicated is not clear if it is pm or am?

Response: Many thanks to the reviewer for noting this. We have added 'am' for clarity into the appropriate section in this table. Please note that this table has been moved to 'supplementary' and is now called 'supplemental table 1' at the editorial team's request.

This section now reads;

ECDC protocol: All patients admitted to the ward before or at 08:00am and not discharged from the ward at the time of survey, including neonates on maternity and paediatric wards, will be included.

Deviations: Only adults ≥18 years old with active malignancy (haematological/ oncological) and or haematopoietic stem cell transplant recipients (allogeneic/ autologous)/ solid organ transplant recipients admitted before or at 08:00am and not discharged from the ward at the time of survey will be included'
